# Diarrhœa and Cholera: Their Origin, Proximate Cause and Cure, through the Agency of the Nervous System, by Means of Ice

**Published:** 1866-08

**Authors:** 


Diarrhœa and Cholera: their Origin, Proximate Cause and Cure, through the Agency of the Nervous System, by Means of Ice. By John Chapman, M.D., M.R.C.P., M.R.C.S. J. B. Lippincott & Co., Philadelphia. 1866.

This is a pamphlet of 57 pages, devoted to the advocacy of ice bags to the spine, as the leading remedial agent in the treatment of cholera.

